# Building transparency and reproducibility into the practice of pharmacoepidemiology and outcomes research

**DOI:** 10.1093/aje/kwae087

**Published:** 2023-05-23

**Authors:** Shirley V Wang, Anton Pottegård

**Affiliations:** Division of Pharmacoepidemiology and Pharmacoeconomics, Brigham and Women’s Hospital, Boston, MA 02120, United States; Department of Medicine, Harvard Medical School, Boston, MA 02115, United States; Clinical Pharmacology, Pharmacy and Environmental Medicine, Department of Public Health, Faculty of Health Sciences, University of Southern Denmark, 5230 Odense M, Denmark

**Keywords:** transparency, reproducibility, real-world evidence, open science, pharmacoepidemiology

## Abstract

Real-world evidence (RWE) studies are increasingly used to inform policy and clinical decisions. However, there remain concerns about the credibility and reproducibility of RWE studies. While there is universal agreement on the critical importance of transparent and reproducible science, the building blocks of open science practice that are common across many disciplines have not yet been built into routine workflows for pharmacoepidemiology and outcomes researchers. Observational researchers should highlight the level of transparency of their studies by providing a succinct statement addressing study transparency with the publication of every paper, poster, or presentation that reports on an RWE study. In this paper, we propose a framework for an explicit transparency statement that declares the level of transparency a given RWE study has achieved across 5 key domains: (1) protocol, (2) preregistration, (3) data, (4) code-sharing, and (5) reporting checklists. The transparency statement outlined in the present paper can be used by research teams to proudly display the open science practices that were used to generate evidence designed to inform public health policy and practice. While transparency does not guarantee validity, such a statement signals confidence from the research team in the scientific choices that were made.

## Introduction

Real-world evidence (RWE) studies are increasingly being used to inform policy and clinical decisions. However, there remain concerns about the credibility of RWE studies. One such area of concern, which has been consistently articulated by various stakeholders,^1^^‑^^5^ is a need for greater transparency and reproducibility in the conduct of database studies.

Biomedical journals and large decision-making organizations are currently working on and may adopt heterogeneous policies and procedures regarding transparency requirements for database studies. However, investigators do not have to wait for mandates to follow transparent research practices. Rather, they can highlight such practices with each paper that they produce.

In this paper, we propose a framework for explicitly stating the level of transparency that a given RWE study has achieved across 5 key domains: (1) protocol, (2) preregistration, (3) data, (4) code-sharing, and (5) reporting checklists.

## Transparency statement for researchers

Researchers can highlight the transparency of their research by providing a succinct statement addressing the 5 domains of study transparency with the publication of every paper, poster, or presentation that reports on a database study—for example, pharmacoepidemiology or health outcomes research.

The proposed transparency statement for researchers is based on the Transparency and Openness Promotion (TOP) guidelines developed a decade ago for journals to encourage more transparent and reproducible scientific practices.^6^ The TOP guidelines have garnered a commitment from over 5000 journals, mostly in the social sciences, to review and implement 8 modular open-science standards with increasing levels of stringency over time as part of the policies and procedures required for publication. Among these journals, there is increasing use of positive reinforcement methods, such as the display of Open Science Badges on publications.^7^^‑^^9^

**Figure 1:**
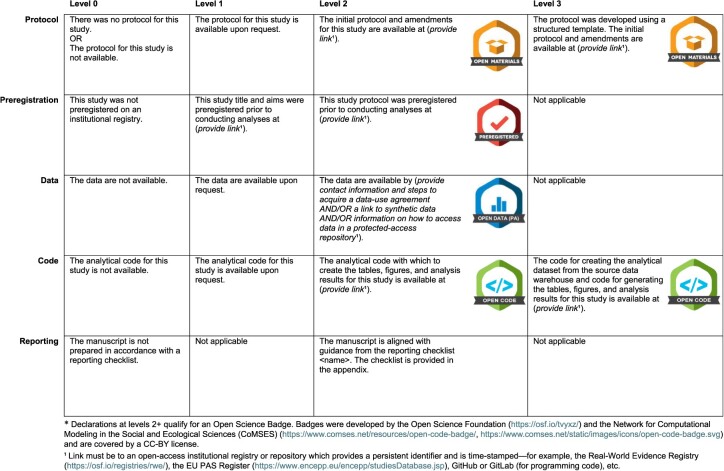
Construction of a 5-piece transparency declaration* for a real-world evidence study.

The TOP guidelines focus on actions and policies that journals can take. However, the uptake of the TOP guidelines in clinical and epidemiology journals has been slow compared with the social sciences, where a heavily publicized “reproducibility crisis” provided an impetus for rapid change.^10^ Therefore, we borrow from the TOP framework and put it in the hands of researchers to declare and display the levels to which they have built transparent and reproducible research practices into each study. Example sentences with which to build a transparency statement are provided in Figure 1.

In the following sections we describe the 5 key domains of a transparency statement, discuss why each domain is important, briefly describe current practices, and suggest how to structure a statement that covers each aspect of study transparency, altogether forming a transparency statement for inclusion in a scientific manuscript.

## Five building blocks for a transparency statement

### Protocol

A well-documented protocol is essential for all database studies, regardless of whether the study is exploratory or confirmatory or whether the goal is to conduct descriptive, predictive, or inferential analyses. A thorough study protocol serves several important purposes, describing the research question, the study design, the data sources, and how study parameters will be measured and analyzed. The study protocol also often serves as a “contract” between lead investigator, collaborators, and study staff. When the scope is agreed upon and documented in the protocol, this not only helps prevent “scope creep” but also facilitates efficient study conduct by providing clear guidance to the analyst. Finally, concerns about fishing or “data dredging” can also be ameliorated by clear documentation and definition of primary analyses in a protocol.

Protocols will, however, often require updates. Working with secondary data that was not collected for research means that there will almost always be unexpected quirks that will necessitate amendments to the original plan outlined in the protocol. Further, unexpected findings might trigger additional analyses, either as exploratory post hoc analyses or as independent subinvestigations in their own right. The fact that a protocol was drafted should never be used as an argument against improving one’s methodology whenever possible or recognizing and pursuing new insights or even new hypotheses in the material. Any such improvements or additions should, however, be transparently argued and documented. Therefore, a good version control system with a clear, contemporaneous record of amendments, including documentation of what, when, and importantly why they were made, is important. Additionally, a log describing the learning that led to amendments not only justifies the changes to the initial protocol but also documents important insights that support future studies in the same database or within the same topic.

While some form of protocol may be used by most investigators, those who review protocols often express that they see a great deal of variation in quality. Further, not all groups within pharmacoepidemiology and outcomes research have established a practice of finalizing study protocols, with a final signoff from all investigators and with documentation of subsequent amendments.

Protocol quality can be raised by using already existing protocol templates that incorporate pharmacoepidemiology good practice guidance. Indeed, researchers in postauthorization studies requested by the European Medicines Agency are already required to use such templates. Recently, 4 existing protocol guidance documents^11^^‑^^14^ were harmonized to incorporate study background/planning/rationale with clear communication of the operational details of implementation that are necessary for reproducibility, which led to the creation of the Harmonized Protocol to Enhance Reproducibility (HARPER),^15^ which was endorsed by the International Society of Pharmacoepidemiology and the International Society for Pharmacoeconomics and Outcomes Research.

*Transparency statement*: If a protocol was not created or is not available, then authors could state so (level 0). If the protocol exists yet is only available upon request, this should be stated (level 1). Note that “available upon request” is not considered sufficient to earn an open protocol badge. If the research team can provide a link to a protocol (including amendments) stored in an open-access registry or open repository that can provide a persistent identifier with time stamps (level 2), they will have earned an Open Science Badge (Figure 1). Note that protocols can be made public outside of the context of preregistration. The research team can attain an even higher level of transparency if their protocol was developed using a recognized structured template such as HARPER^15^ (level 3).

### Preregistration

Public registration of database studies has several benefits for the research enterprise as a whole. The key value of preregistration is publishing a time-stamped protocol. A repository of conducted database studies could reduce research waste and publication bias.^16^ Further, the knowledge that a preregistered protocol will be turned into a public document will encourage a more thorough and thoughtful discussion within the investigative team about planning and documenting analyses.

That said, preregistration is not of equal importance for all database studies. For studies that aim to evaluate a hypothesis about a treatment effect, preregistration of a well-documented protocol prior to conduct of inferential analysis can help to address concerns about results-driven analyses or selective presentation of findings.^17^ False statements are always possible but may be deterred if research teams are required to sign off on a public attestation of registration prior to inferential analyses. For descriptive, exploratory, or predictive studies, preregistration is less critical. For these types of studies, however, registration remains a means for promoting open science, by providing a stable repository for publicly shared protocols and making research more accessible. Opponents of preregistration may fear being “scooped,” may fear that preregistration of a protocol may interfere with publication, or may fear that preregistration may stifle scientific discovery if results from the initial plan are held to have higher value than unplanned results from analyses that are deviations from the protocol. However, investigators who fear being scooped may choose a study registration site with an embargo feature that allows the date-/time-stamped material to be preserved in a lockbox until the embargo date has passed. With medical journals increasingly supporting publication of preprints, a preregistered protocol is unlikely to interfere with future publication and can be useful supplementary material to reference. For those who fear stifling of scientific discovery by registering a protocol, we refer to the previous section, where we discussed the importance of a contemporaneous record of amendments. Again, we emphasize that a preregistered protocol does not mean that no changes may be made or that results obtained after deviations to the plan should be ignored. In the end, the protocol should provide a road map from the point at which the investigators started to the point at which they ended.

*Transparency statement*: If the study was not preregistered, then authors could state so (level 0). If the research team did not include a protocol in their registration (eg, preregistration provided only title, aims, and summary) (level 1), they may state so. If they registered a full study protocol prior to conducting analyses in an open-access registry or repository that can provide a persistent identifier with time stamps (level 2), they will have earned an Open Science Badge (Figure 1).

### Data

Most database studies use individual-level patient health information such as insurance claims or electronic health record data. While researchers are able to access these data through strict data-use agreements, legal restrictions in these agreements will generally prevent sharing of analytical data or derivates thereof. However, there are a couple of alternatives to publicly sharing data for researchers who work with protected-access health-care data. Researchers could provide guidance for other investigators on how to access the source databases underlying their work. Importantly, such guidance must include details on how to contact and contract with the data holder(s), which version/release of the data was accessed, and/or the date on which that data extraction was performed. While this is often prohibited for database studies, when data-use contracts permit, analytical datasets could be stored and shared via protected-access data repositories.^18^ In addition, or alternatively, researchers could provide synthetic data^19^ that protects patient privacy, retains the statistical properties of the original data, and facilitates testing and use of shared code. Importantly, any data that is shared must follow FAIR principles (findable, accessible, interoperable, reusable), including metadata about how to use the shared materials, a persistent unique identifier, clear access processes, and procedures that account for data protection and data privacy requirements, among other characteristics.^20^

*Transparency statement*: If data are not available, then authors could state so (level 0). If data are only available upon request, this should be stated (level 1). As before, stating “available upon request” is not considered sufficient to earn an Open Science Badge. If the research team provides a link to deidentified data stored in an open access repository OR if they provide an appendix with detailed information on contacts, contracting process, and version/ETL [extract, transform, and load] OR if they provide access to a synthetic dataset along with analysis code (level 2), they will have earned an Open Science Badge for protected-access data (Figure 1). Without these details (ie, when only referencing the data holder by vendor name), it will in most cases be impossible to recreate that data request and therefore impossible to reproduce the analysis.

### Code-sharing

While a well-documented protocol is central to the transparency and reproducibility of a study, computational reproducibility generally further requires the sharing of data with analytical code and other supporting materials such as a “readme” file, data dictionary, and protocol. Such materials provide the steps needed to exactly reproduce the registered study.

Sharing of study materials for database studies, however, is currently limited. First, it requires considerable effort to maintain analytical code so that it is understandable for investigators outside the project or organization. Similarly, it can be demanding to document the necessary metadata. However, transparency is not achieved by posting a link to an unorganized dump of materials, where relevant information is difficult to find or interpret. As an example, sharing long scripts of analytical code without clear annotation of the decisions that are being implemented gives a false sense of transparency without any real impact on reproducibility and ability to evaluate study quality. Git can be a useful tool for creating reproducible code workflows with a well-documented version-control system.^21^ Second, some may consider their research procedures (eg, software code, algorithms) intellectual property that they would not want in the public domain without some form of attribution or recognition. Furthermore, some data vendors do not permit public sharing of code that could reveal the underlying data model of protected-access data. The effort to create and share well-documented research materials will, however, arguably benefit both the researchers and consumers of their work. Algorithms, code, and other materials can be citable resources, with high-quality, findable, and interpretable materials being cited more than data dumps.^20^ Importantly, the Open Science Framework registries are linked to researcher ORCID identifiers and provide digital object identifiers (DOIs) to facilitate identification and citation of posted study materials. Accessible research materials will help database researchers learn and build from each others’ work, thus reducing waste and accelerating growth and innovation.

*Transparency statement:* If analytical code is not available, then authors could state so (level 0). If the code is only available upon request, this should be stated (level 1). Stating that analytical code is “available upon request” is not sufficient to earn an Open Science Badge. If the research team can provide a link to analytical code that at minimum creates tables, figures, and analysis results from a derived analytical dataset in an open access repository (eg, GitHub) (level 2), they will have earned an Open Science Badge (Figure 1). The research team can attain an even higher level of transparency if they also share code or workflows used to generate the derived analytical dataset from source data warehouses (level 3). Importantly, sharing code comes with the responsibility of sharing well-structured scripts with sufficient annotation/comments for it to be digestible by other researchers.

### Reporting checklists

The purpose of publications and reports is to communicate research findings. Unlike protocols, they include key points, results, and interpretation. Research reporting checklists increase the value of research communications by providing guidance on the most critical elements that must be included. Such checklists can be used after conducting the study to create an outline for drafting a clearly written paper, or in the very early phases of research to quickly outline and evaluate a research plan for discussion with an advisor, collaborator, or decision-maker before proceeding with development of a full protocol.

Several checklists are available to support the reporting of RWE studies, and these reporting checklists are also required by many journals. The Strengthening the Reporting of Observational Studies in Epidemiology (STROBE)^22^ checklist is one of the most well recognized. However, within subdisciplines of observational studies, there are substantive differences in the types of information on methodology that are crucial to report. To cover a broad base, some of the items in STROBE are necessarily generic and/or of less relevance for database studies (eg, provide reasons for nonparticipation). Other items that are highly relevant are not addressed (eg, use of a design diagram to provide clarity on temporality of assessment windows). An alternative to STROBE is the Reporting of Studies Conducted using Observational Routinely Collected Health Data Statement for Pharmacoepidemiology (RECORD-PE),^23^ a reporting checklist that builds off of STROBE and is specifically tailored for database studies.

*Transparency statement*: If the manuscript was not prepared in accordance with a reporting checklist, this could be stated (level 0). If the research team used a relevant reporting checklist and they share the checklist with the publication (level 2), they will have earned the open science reporting badge (Figure 1). In the absence of specific requirements by a journal, the RECORD-PE checklist should be preferred.

**Figure 2:**
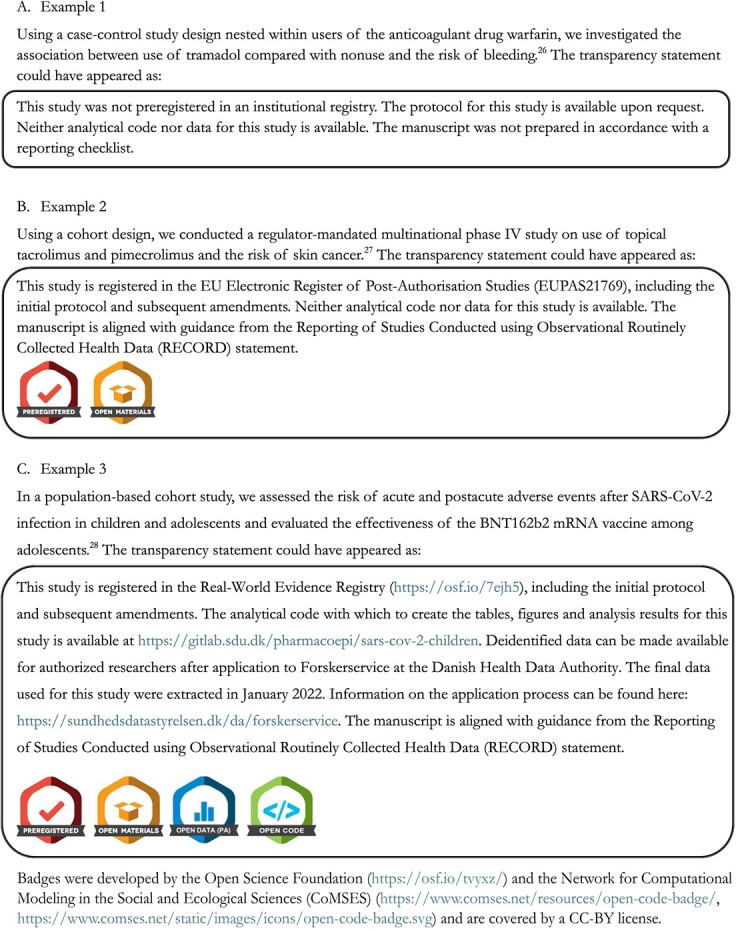
Example transparency statements.

## Examples

Here, we provide 3 “transparency statements” as examples (Figure 2). They are constructed on the basis of 3 previous papers from our own research teams and are selected to exemplify the evolution of transparent and reproducible research practices over time in our own work. Notably, even the most recent example does not attain full transparency according to the present framework. Thus, these examples also highlight areas to focus on to build greater transparency into our pharmacoepidemiology and health outcomes research.

These examples highlight implementation of proposed wording to cover each of the 5 domains. However, this language—the exact wording of the transparency statement—can and should be adjusted to the individual case. For example, the statement for examples 2 and 3 combines the statement on preregistration with the statement on protocol availability, as the two are interlinked for these particular papers. This may not always be the case. Similarly, highlighting transparency domains not covered by a given paper (the domains at “level 0”) could be considered optional.

## Discussion

While there is universal agreement on the critical importance of transparent and reproducible science, the building blocks of open science practice that are universal across disciplines have not yet been built into routine workflows for many pharmacoepidemiology and outcomes researchers.

The ideas that are summarized in the proposed transparency statement are not new. Regulators, health technology assessors, and other decision-making organizations either have or are currently considering imposing preregistration, protocol, and other requirements for certain types of database studies submitted to their organizations.^5^^,^^24^^‑^^26^ Clinical and epidemiology journals as well as professional conferences are also considering policies, processes, standards, or mandates to improve transparency and reproducibility. At the same time, there are ongoing discussions about adoption of positive reinforcement methods that have been successful in other fields,^9^ such as the display of Open Science Badges on publications, posters, and abstracts. Display of such badges prompts discussion and can lead to a cultural shift by inspiring others to earn similar recognition. Future potential incentives can easily be envisioned (eg, peer reviewers’ being more likely to accept review tasks for papers with badges) or other types of promotion (eg, via social media or open science metrics analogous to the Hirsch index (*h*-index) for papers that have earned badges). Development of processes and infrastructure to support follow-through on declarations, such as recent new policies on data, protocol, and code-sharing from funders like the National Institutes of Health^27^ and the Patient-Centered Outcomes Research Institute,^28^ are important, as a declaration does not guarantee action, and it has been observed that in spite of modest increases in declarations of data- or code-sharing for medical research in recent years, actual sharing of such materials has not necessarily increased.^29^

That said, we do not have to wait for new incentives, mandates, or policies to be developed across different stakeholder organizations. The transparency statements outlined in the present paper can be used by research teams to proudly display the open science practices that were used to generate evidence designed to inform public health policy and practice. Such a statement signals confidence from the research team in the scientific choices that were made and indicates that the research team is not simply asking the evidence consumer to trust that they know what they are doing; rather they are willing to show their work, to show how the evidence was generated, and make it possible for the results to be reproduced or replicated.

It is important to note that transparency and validity are often conflated. While the statement proposed in this paper is focused on transparency, it is possible to produce highly transparent research that is intractably biased. Conversely, one can also produce valid and robust causal inferences from database studies without being transparent about how that evidence was generated. Importantly, we see a move toward greater transparency as a move toward validity not because transparency equals validity but because transparency enables researchers, reviewers, and clinical, regulatory, and coverage decision-makers to better assess validity. Transparency on the 5 building blocks will provide evidence consumers with the materials needed to differentiate the highly valid database studies whose evidence they would want to use to inform decision-making from the flawed studies whose evidence they want to disregard.

Akin to the transformation among trialists 2 decades ago, the power to realize a future where RWE is recognized as consistently transparent, reproducible, and fit for decision-making lies in the hands of current and future researchers. We both hope and expect to see transparency statements and Open Science Badges become ubiquitous in pharmacoepidemiology and outcomes research over the years to come.

## Data Availability

This article does not involve data analysis. Therefore, there is no protocol to preregister, nor is there software code or data to share. There are no applicable reporting guidelines for this article. The sources of all Open Science Badges are provided in the figure footnotes.

## References

[1] US Food and Drug Administration . *Framework for FDA’s Real-World Evidence Program*. US Food and Drug Administration; 2018. Accessed January 31, 2019. https://www.fda.gov/media/120060/download

[2] European Medicines Agency . *The ENCePP Code of Conduct For Scientific Independence and Transparency in the Conduct of Pharmacoepidemiological and Pharmacovigilance Studies*. (Publication no. EMA/929209/2011). European Medicines Agency; 2019. Accessed April 14, 2020. https://encepp.europa.eu/document/download/e504e741-1813-4327-85d5-0f7ac8214ca1_en?filename=ENCePPCodeofConduct.pdf

[3] Makady A , Ham RT, de Boer A, et al. Policies for use of real-world data in health technology assessment (HTA): a comparative study of six HTA agencies. *Value Health*. 2017;20(4):520-532. 10.1016/j.jval.2016.12.003 28407993

[4] Berger ML , Sox H, Willke RJ, et al. Good practices for real-world data studies of treatment and/or comparative effectiveness: recommendations from the joint ISPOR-ISPE Special Task Force on real-world evidence in health care decision making. *Pharmacoepidemiol Drug Saf*. 2017;26(9):1033-1039. 10.1002/pds.4297 28913966 PMC5639372

[5] Hampson G , Towse A, Dreitlein WB, et al. Real-world evidence for coverage decisions: opportunities and challenges. *J Comp Eff Res*. 2018;7(12):1133-1143. 10.2217/cer-2018-0066 30411972

[6] Center for Open Science . TOP Factor. Accessed November 4, 2022. https://www.cos.io/initiatives/top-guidelines

[7] Center for Open Science . What are Open Science Badges? Accessed October 20, 2022. https://www.cos.io/initiatives/badges

[8] CoMSES Network . Open Code Badge. Accessed January 10, 2023. https://www.comses.net/resources/open-code-badge/

[9] Kidwell MC , Lazarevic LB, Baranski E, et al. Badges to acknowledge open practices: a simple, low-cost, effective method for increasing transparency. *PLoS Biol*. 2016;14(5):e1002456. 10.1371/journal.pbio.1002456 27171007 PMC4865119

[10] Korbmacher M , Azevedo F, Pennington CR, et al. The replication crisis has led to positive structural, procedural, and community changes. *Commun Psychol*. 2023;1(1):3. 10.1038/s44271-023-00003-2 39242883 PMC11290608

[11] Wang SV , Pinheiro S, Hua W, et al. STaRT-RWE: structured template for planning and reporting on the implementation of real world evidence studies. *BMJ*. 2021;372:m4856. 10.1136/bmj.m4856 33436424 PMC8489282

[12] European Medicines Agency . *Guidance for the Format and Content of the Protocol of Non-Interventional Post-Authorisation Safety Studies.* (Publication no. EMA/623947/2012). Updated September 26, 2012. Accessed January 4, 2022. https://www.ema.europa.eu/en/documents/other/guidance-format-content-protocol-non-interventional-post-authorisation-safety-studies_en.pdf

[13] ISPE . Guidelines for good pharmacoepidemiology practices (GPP). Initially issued: 1996, Revision 1: August 2004; Revision 2: April 2007, Revision 3: June 2015. https://www.pharmacoepi.org/resources/policies/guidelines-08027/

[14] National Evaluation System for Health Technology . *National Evaluation System for health Technology Coordinating Center (NESTcc) Methods Framework: A Report of the Methods Subcommittee of the NEST Coordinating Center—An initiative of MDIC*. February 2020. Accessed September 28, 2023. https://nestcc.org/data-quality-and-methods/

[15] Wang SV , Pottegard A, Crown W, et al. HARmonized Protocol Template to Enhance Reproducibility of hypothesis evaluating real-world evidence studies on treatment effects: a good practices report of a joint ISPE/ISPOR task force. *Pharmacoepidemiol Drug Saf*. 2022;32(1):44-55. 10.1002/pds.5507 36215113 PMC9771861

[16] Williams RJ , Tse T, Harlan WR, et al. Registration of observational studies: is it time? *CMAJ.* 2010;182(15):1638-1642. 10.1503/cmaj.092252 20643833 PMC2952011

[17] Orsini LS , Monz B, Mullins CD, et al. Improving transparency to build trust in real-world secondary data studies for hypothesis testing—why, what, and how: recommendations and a road map from the real-world evidence transparency initiative. *Pharmacoepidemiol Drug Saf*. 2020;29(11):1504-1513. 10.1002/pds.5079 32924243

[18] Kurz X , Perez-Gutthann S, ENCePP Steering Group. Strengthening standards, transparency, and collaboration to support medicine evaluation: ten years of the European Network of Centres for Pharmacoepidemiology and Pharmacovigilance (ENCePP). *Pharmacoepidemiol Drug Saf*. 2018;27(3):245-252. 10.1002/pds.4381 29327451 PMC5873428

[19] Center for Open Science. Real World Evidence Registry . Accessed October 20, 2022. https://osf.io/registries/rwe/discover

[20] Kuntz RE , Antman EM, Califf RM, et al. Individual patient-level data sharing for continuous learning: a strategy for trial data sharing. (Discussion paper). July 1, 2019. Accessed September 28, 2023. https://nam.edu/individual-patient-level-data-sharing-for-continuous-learning-a-strategy-for-trial-data-sharing/10.31478/201906b PMC8406566 34532668

[21] Gonzales A , Guruswamy G, Smith SR. Synthetic data in health care: a narrative review. *PLoS Digit Health*. 2023;2(1):e0000082. 10.1371/journal.pdig.0000082 36812604 PMC9931305

[22] García-Closas M , Ahearn TU, Gaudet MM, et al. Moving toward findable, accessible, interoperable, reusable practices in epidemiologic research. *Am J Epidemiol*. 2023;192(6):995-1005. 10.1093/aje/kwad040 36804665 PMC10505418

[23] Weberpals J , Wang SV. The FAIRification of research in real-world evidence: a practical introduction to reproducible analytic workflows using Git and R. *Pharmacoepidemiol Drug Saf*. 2024;33(1):e5740. 10.1002/pds.5740 38173166

[24] von Elm E , Altman DG, Egger M, et al. The Strengthening the Reporting of Observational Studies in Epidemiology (STROBE) statement: guidelines for reporting observational studies. *PLoS Med*. 2007;4(10):e296. 10.1371/journal.pmed.0040296 17941714 PMC2020495

[25] Langan SM , Schmidt SA, Wing K, et al. The reporting of studies conducted using observational routinely collected health data statement for pharmacoepidemiology (RECORD-PE). *BMJ*. 2018;363:k3532. 10.1136/bmj.k3532 30429167 PMC6234471

[26] Pottegard A , Meegaard PM, Holck LH, et al. Concurrent use of tramadol and oral vitamin K antagonists and the risk of excessive anticoagulation: a register-based nested case-control study. *Eur J Clin Pharmacol*. 2013;69(3):641-646. 10.1007/s00228-012-1363-x 22847619

[27] Arana A , Pottegard A, Kuiper JG, et al. Long-term risk of skin cancer and lymphoma in users of topical tacrolimus and pimecrolimus: final results from the extension of the cohort study Protopic Joint European Longitudinal Lymphoma and Skin Cancer Evaluation (JOELLE). *Clin Epidemiol*. 2021;13:1141-1153. 10.2147/CLEP.S331287 35002327 PMC8721027

[28] Kildegaard H , Lund LC, Hojlund M, et al. Risk of adverse events after covid-19 in Danish children and adolescents and effectiveness of BNT162b2 in adolescents: cohort study. *BMJ*. 2022;377:e068898. 10.1136/bmj-2021-068898 35410884 PMC8995669

[29] US Food and Drug Administration . *Considerations for the Use of Real-World Data and Real-World Evidence to Support Regulatory Decision-Making for Drug and Biological Products: Guidance for Industry*. (Publication no. FDA-2021-D-1214). US Food and Drug Administration; 2023. Accessed October 20, 2022. https://www.fda.gov/regulatory-information/search-fda-guidance-documents/considerations-use-real-world-data-and-real-world-evidence-support-regulatory-decision-making-drug

[30] National Institute for Health and Care Excellence . NICE real-world evidence framework. June 23, 2022. Accessed October 20, 2022. https://www.nice.org.uk/corporate/ecd9/chapter/overview 10.57264/cer-2023-0135 PMC10690376 37855246

[31] National Institutes of Health . 2023 NIH Data Management and Sharing Policy. March 1, 2024. Accessed September 28, 2023. https://oir.nih.gov/sourcebook/intramural-program-oversight/intramural-data-sharing/2023-nih-data-management-sharing-policy

[32] Patient-Centered Outcomes Research Institute . PCORI’s policy for data management and data sharing. September 7, 2018. Updated September 1, 2023. Accessed January 2, 2024. https://www.pcori.org/about/governance/policy-data-management-and-data-sharing

[33] Hamilton DG , Hong K, Fraser H, et al. Prevalence and predictors of data and code sharing in the medical and health sciences: systematic review with meta-analysis of individual participant data. *BMJ*. 2023;382:e075767. 10.1136/bmj-2023-075767 37433624 PMC10334349

